# Genome-Wide Analysis of Antigen 43 (Ag43) Variants: New Insights in Their Diversity, Distribution and Prevalence in Bacteria

**DOI:** 10.3390/ijms24065500

**Published:** 2023-03-13

**Authors:** Valentin Ageorges, Ivan Wawrzyniak, Philippe Ruiz, Cédric Bicep, Mohamed A. Zorgani, Jason J. Paxman, Begoña Heras, Ian R. Henderson, Sabine Leroy, Xavier Bailly, Panagiotis Sapountzis, Eric Peyretaillade, Mickaël Desvaux

**Affiliations:** 1 INRAE, UCA, UMR0454 MEDIS, 63000 Clermont-Ferrand, France; 2 UCA, CNRS, UMR6023 LMGE, 63000 Clermont-Ferrand, France; 3 Department of Biochemistry and Chemistry, La Trobe Institute for Molecular Science, La Trobe University, Bundoora, VIC 3086, Australia; 4 Institute for Molecular Biosciences, University of Queensland, St. Lucia, QLD 4067, Australia; 5 INRAE, UCA, VetAgro Sup, UMR0346 EPIA, 63122 Saint Genes Champanelle, France

**Keywords:** *Escherichia coli*, protein secretion, autotransporter, Type V protein secretion system (T5SS), phylogeny, gene diversity, bacterial pathogens, cell surface protein, aggregation, bacterial colonisation and infection and infection

## Abstract

Antigen 43 (Ag43) expression induces aggregation and biofilm formation that has consequences for bacterial colonisation and infection. Ag43 is secreted through the Type 5 subtype “a” secretion system (T5aSS) and is a prototypical member of the family of self-associating autotransporters (SAATs). As a T5aSS protein, Ag43 has a modular architecture comprised of (i) a signal peptide, (ii) a passenger domain that can be subdivided into three subdomains (SL, EJ, and BL), (iii) an autochaperone (AC) domain, and (iv) an outer membrane translocator. The cell-surface SL subdomain is directly involved in the “Velcro-handshake” mechanism resulting in bacterial autoaggregation. Ag43 is considered to have a ubiquitous distribution in *E. coli* genomes and many strains harbour multiple *agn43* genes. However, recent phylogenetic analyses indicated the existence of four distinct Ag43 classes exhibiting different propensities for autoaggregation and interactions. Given the knowledge of the diversity and distribution of Ag43 in *E. coli* genomes is incomplete, we have performed a thorough in silico investigation across bacterial genomes. Our comprehensive analyses indicate that Ag43 passenger domains cluster in six phylogenetic classes associated with different SL subdomains. The diversity of Ag43 passenger domains is a result of the association of the SL subtypes with two different EJ-BL-AC modules. We reveal that *agn43* is almost exclusively present among bacterial species of the Enterobacteriaceae family and essentially in the *Escherichia* genus (99.6%) but that it is not ubiquitous in *E. coli*. The gene is typically present as a single copy but up to five copies of *agn43* with different combinations of classes can be observed. The presence of *agn43* as well as its different classes appeared to differ between *Escherichia* phylogroups. Strikingly, *agn43* is present in 90% of *E. coli* from E phylogroup. Our results shed light on Ag43 diversity and provide a rational framework for investigating its role in *E. coli* ecophysiology and physiopathology.

## 1. Introduction

Autotransporters (ATs) belonging to the Type 5 subtype “a” secretion system (T5aSS) can be found in lipopolysaccharide-containing diderm bacteria (archetypal Gram-negative bacteria) [1,2,3]. These modular proteins consist of three main domains: (i) an N-terminal signal peptide (SP), (ii) a central passenger domain, and (iii) a C-terminal β-barrel translocator [4,5]. While targeting to the inner membrane (IM) and while export via the Sec translocase is facilitated by the SP, the passenger domain is secreted across the outer membrane (OM) through a β-barrel pore formed by the translocator. As an effector, the AT passenger domain can have a range of different functions, which have been used to classify ATs into distinct functional categories. One such category is the bacterial surface bound self-associating ATs (SAATs) [4,6,7], whose primary function is to interact and associate with SAATs on neighbouring bacterial surfaces. As a result of this homotypic interaction, bacterial cells aggregate together and sediment [7,8]. This autoaggregation phenomenon mediates crucial processes related to bacterial physiology, ecophysiology, and/or physiopathology. For example, it can influence biofilm formation, gather siblings in mixed bacterial communities, and/or affect bacterial virulence and infection [7,9].

Prototypical members of SAATs include AIDA (adhesin involved in diffuse adherence), TibA (toxigenic invasion locus b), and Ag43 (antigen 43) [6,7]. The SAATs belong to the family of AIDA-type AT and, like many other AT adhesins, the passenger is predicted to be composed of right-handed β-helix structures (also called Type 1 AT passenger structure) [4,6]. However, the SAATS are distinct from other adhesins, such as UpaB (uropathogenic *E. coli* autotransporter adhesin B) or EhaB (enterohemorrhagic *Escherichia coli* autotransporter B), which do not trigger autoaggregation but specifically bind some extracellular matrix molecules [6,10]. Some other ATs promote autoaggregation but not as their primary function [4], e.g., Hap (*Haemophilus influenzae* adhesion and penetration protein) is primarily a serine protease AT with adhesive and aggregative properties harboured in its C-terminal region [11], and AutA (autotransporter A) appears to mediate aggregation through direct or indirect interaction with extracellular DNA [12]. While EhaA has been suspected of being a SAAT because of its autoaggregative properties [13,14], the most recent phylogenetic analysis placed it into another subgroup of adhesins that promote both biofilm formation and binding to host cells, together with MisL (membrane insertion and secretion protein L) [15] and IcsA (intracellular spread protein A) [16]. More recently, a number of additional putative SAATs have been uncovered, namely YapA, YcgV, YapC and Ypj, as well as the more distantly related RadD [6]. These new and putative SAATs await further functional characterisation.

**Figure 1:**
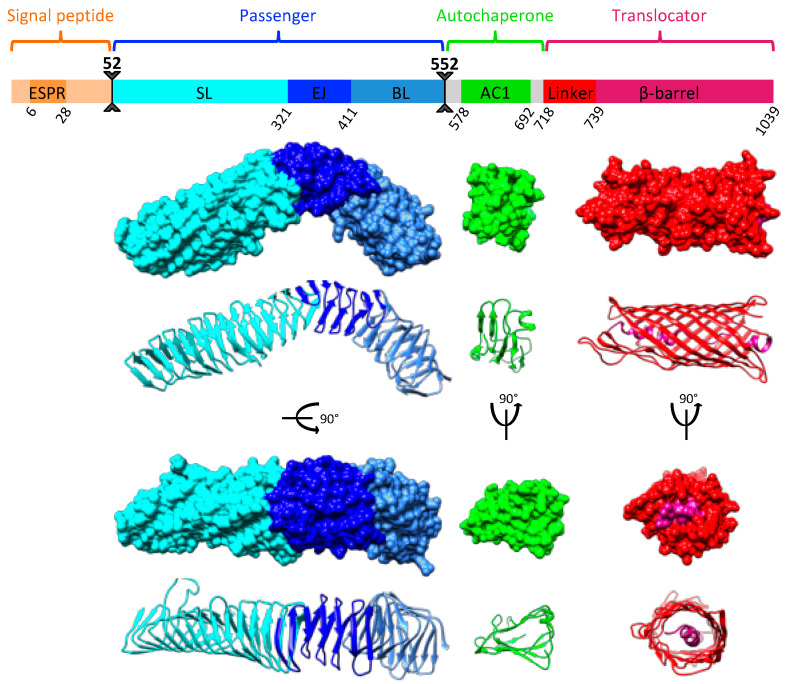
Modular organisation of the SAAT Ag43. The Ag43 ^C3^ from UPEC CFT073 is presented. It is composed of an N-terminal SP exhibiting a conserved ESPR (IPR024973), a passenger domain corresponding to the effector region responsible for the autoaggregation function, a structurally conserved AC1 domain involved in folding, and a C-terminal translocator comprising a α-helical linker region and a β-barrel. The SP is processed between amino acid position 51 and 52 and the passenger is cleaved off from the AC1 and translocator regions between amino acid 551 and 552. The 3D structures are given as surface (upper part) and ribbon diagrams (lower part), which were generated from UCSF Chimera [19]. The passenger and AC1 correspond to the resolved structure of Ag43 ^C3^ from UPEC CFT073 (PDB: 4KH3, 7KOB) [20,21], whereas the translocator was modelled with Phyre2 [22] with the translocator from AIDA-I (PDB: 4MEE) [23] used as a template.

Ag43 is considered to be the major phase-variable outer membrane protein of *E. coli* and the most well investigated SAAT [17,18]. Besides the shared and common structural features of ATs secreted by the T5aSS, Ag43 exhibits some additional conserved domains (Figure 1).

The SP harbours a highly conserved extended region (ESPR) that regulates the rate of export through the Sec translocase at the IM. This allows the formation of a competent passenger–translocator complex in the OM, which is required for efficient passenger transport to the bacterial cell surface [24,25]. A structurally conserved autochaperone domain of type 1 (AC1) is present between the passenger and the translocator. It is considered to be essential for the folding of β-helical passengers [26,27]. Following Ag43 production as a preproprotein, the SP is cleaved off after export across the IM by a type 1 signal peptidase and the passenger is cleaved between the AC1 and translocator after secretion across the OM by an unidentified protease (Figure 1). The Ag43 passenger can subsequently remain associated with the bacterial cell surface via non-covalent interactions [4]. While Ag43 can be naturally found in glycosylated and unglycosylated forms depending on the *E. coli* strains [28], the endogenous glycosyltransferases at play remain unknown and the biological relevance of glycosylation in Ag43 is still unclear. In fact, O-glycosylation is not a prerequisite for Ag43 function, including autoaggregation, bacterial adhesion, and biofilm formation, which rely on distinct mechanisms [28]. Ag43 was originally classified in two subfamilies, SF-I and SF-II, but a recent phylogenetic network analysis focusing on the passenger revealed four distinct functional classes, i.e., C1, C2, C3, and C4 [29]. The crystal structure of the Ag43 ^C3^ passenger from UPEC CFT073 was solved and revealed to form a twisted L-shape β-helical conformation, which was shown to be a requirement for the handshake self-association [21]. Further structural and phylogenetic analyses demonstrated the L-shaped Ag43 passengers could be subdivided into SL (stem of the L shape), EJ (elbow joining), and BL (bottom of the L shape) subdomains, each belonging to two distinct subtypes. This variation through domain shuffling is responsible for the occurrence of the four distinct Ag43 classes [20,29].

Ag43 is considered to have a quite ubiquitous distribution across *E. coli* strains and *agn43* is often present in multiple copies in a single strain [17,30]. While the presence of *agn43* appeared to be more frequent among UPEC and diarrheagenic *E. coli* (DEC) than commensal isolates [30,31], this must be interpreted with caution as it could simply reflect a bias introduced by the greater availability of pathogenic *E. coli* genomes compared to non-pathogenic ones [18]. With this in mind, we performed comprehensive data mining to uncover additional Ag43 homologues and assess their modular architecture, and to reinvestigate the phylogenetic diversity and distribution of this important SAAT.

## 2. Results

### 2.1. Phylogenetic Analyses of Ag43 Passenger Domains

**Figure 2:**
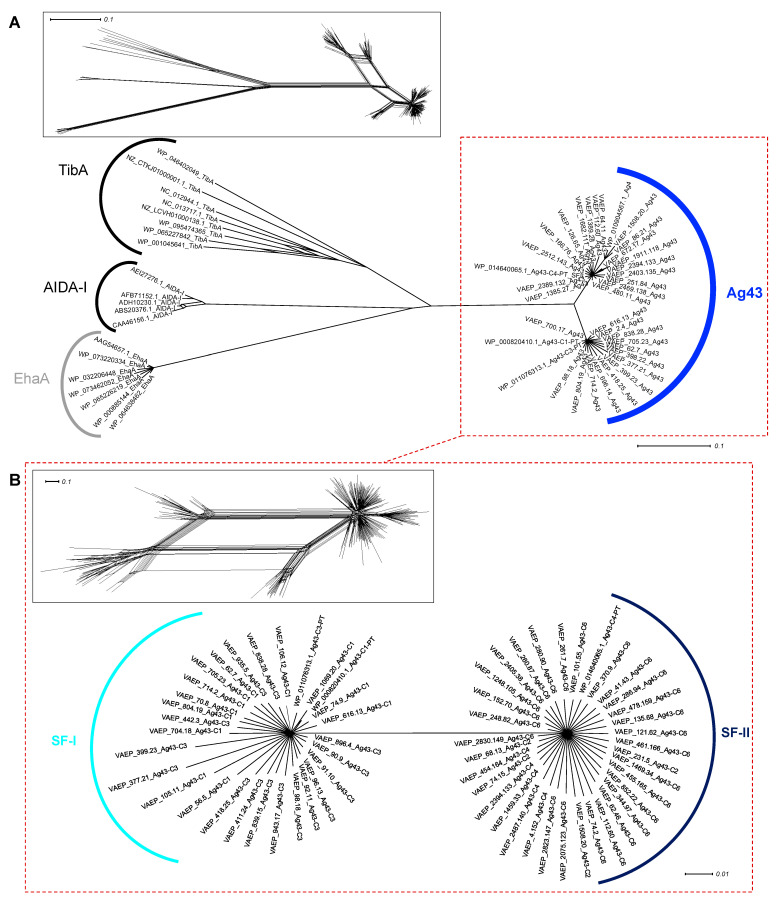
Phylogenetic analysis of Ag43 and other SAATs. Trees were generated using SplitsTree and the inserts represent the trees before applying bootstrapping and confidence threshold. (**A**) Full-length amino acid sequences of Ag43 respective to other SAATs, namely TibA and AIDA-I [4,7,32]; EhaA was used as an outgroup (in grey). (**B**) Phylogenetic analyses of Ag43, highlighting the two subfamilies previously reported, namely SF-I and SF-II [18]. For legibility, sequences that were identified but not presented here are available in supplementary Table S1. Scale bars represent the evolutionary distance, i.e., the number of substitutions per site.

Following our data mining approach to identify Ag43 homologues in Bacteria, 9988 Ag43 sequences were recovered, including 7336 full-length sequences corresponding to 1549 unique sequences for the passenger domain; this substantially expanded the previous dataset of 106 Ag43 sequences [13,18,29]. After clustering with a threshold set at 98% identity, a dataset of 247 sequences representative of the diversity of Ag43 was generated for further phylogenetic studies (supplementary Table S1). To confirm they belong to the Ag43 family, and not to other SAATs, we performed a phylogenetic network analysis, which demonstrated that all of the newly discovered Ag43 clustered separately from TibA and AIDA-I (Figure 2A). Focusing on Ag43, the phylogenetic network analysis first suggested an ambiguity in the relatedness patterns among the Ag43 homologues as illustrated by the diamond shape of the network, most certainly related to domain shuffling (Figure 2B). However, bootstrapping analysis with a stringent confidence threshold (90%) revealed two major clusters corresponding to a dichotomous grouping of Ag43 as previously reported [18], dividing the predicted proteins into the two subfamilies SF-I and SF-II.

**Figure 3:**
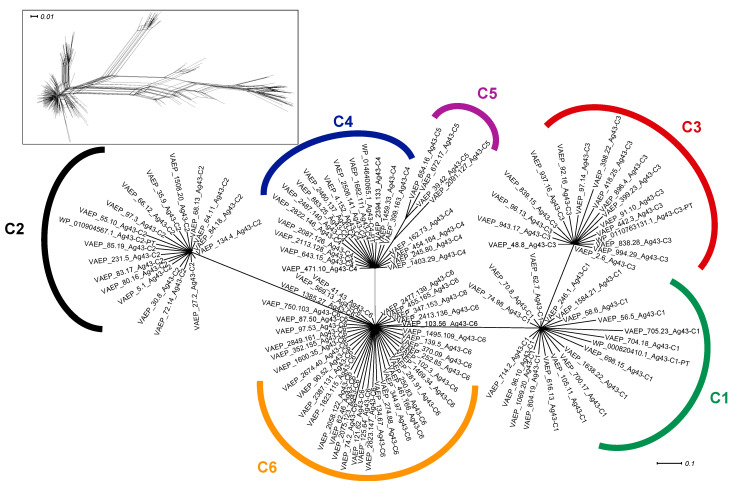
Phylogenetic analysis of the passenger domain of Ag43. Trees were generated using SplitsTree; the insert represents the trees before applying bootstrapping and confidence threshold. Ag43 groups into six different classes. For legibility, sequences that were identified but have not been included here are available in Table S1. Scale bars represent the evolutionary distance.

Subsequently, split decomposition analyses were applied to the functional passenger domain of Ag43 only (Figure 3). A diamond shape network was obtained in the first instance, but bootstrapping analysis resolved the splits and revealed the existence of six classes of Ag43 passengers. Previous network analysis on Ag43 (using a much smaller dataset) evidenced the existence of four main passenger classes supported by robust splits but with a strong ambiguity in their relatedness patterns and no specific divergence between C1 and C4 [29]. However, our present analysis (increasing the dataset from 106 to 247 non-redundant sequences) alleviated the uncertainties between C3 and C2, C1 and C4, as well as C2 and C4, since it clearly indicated direct links only between: (i) C4 and the newly identified C5, (ii) C2 and the newly identified C6, (iii) C1 and C3, as well as (iv) C6 and C1. Indeed, the original SF-I subfamily can be divided into C1 and C3, whereas SF-II can be divided into C2, C4, C5, and C6 (Figure 2B and Figure 3).

As previously reported [29], there is often (but not always) a relationship between clustering and the size of Ag43 passengers, which ranges from between 500 amino acids for C1 and C3, and 430 amino acids for the four remaining classes (supplementary Table S1). While C1 to C4 harbour previously investigated Ag43 homologues, namely from *E. coli* K12 MG1655 (UPI00003B25EE), enterohaemorrhagic *E. coli* (EHEC) O157:H7 EDL933 (UPI00000D07E5), uropathogenic *E. coli* (UPEC) CFT073 (UPI00000E49D2), and enterotoxigenic *E. coli* (ETEC) O78:H11 H10407, respectively [29], no Ag43 homologues from C5 and C6 have been functionally or structurally characterised to date.

**Figure 4:**
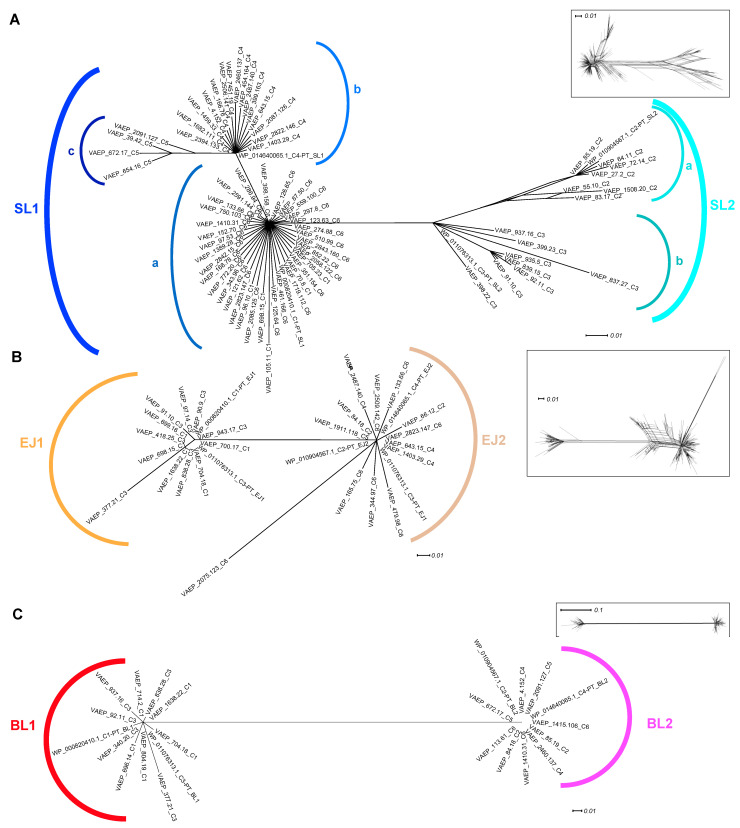
Phylogenetic analysis of the Ag43 passenger subdomains SL, EJ, and BL. Trees were generated using SplitsTree; inserts represent the trees before applying bootstrapping and confidence threshold. (**A**) SL subdomain from the Ag43 passenger respective to the types previously reported, namely SL1 and SL2 [29]; SL1 further split into subtypes a, b, and c, and SL2 into subtypes a and b. (**B**) EJ subdomain from the Ag43 passenger respective to the subtypes previously reported, namely EJ1 and EJ2 [29]. (**C**) BL subdomain from the Ag43 passenger respective to the subtypes previously reported, namely BL1 and BL2 [29]. For legibility, sequences that were identified but have not been included here are available in Table S1. Scale bars represent the evolutionary distance.

Given the passenger domain is composed of three different subdomains [20,21,29], further phylogenetic network analyses were performed on each subdomain (Figure 4). While two robust clusters of SL sequences of type 1 and 2 were still unambiguously differentiated, it further appeared that SL1 and SL2 could be further divided into subtypes. SL1 split into clusters represented by subtypes a, b, and c, whereas SL2 could be differentiated into two subtypes, a and b (Figure 4A).

SL1a corresponds to the SL1 originally identified in Ag43 ^C1-PT^ and contains Ag43 proteins classified as C1 as well as the newly identified C6. SL1b exclusively contains Ag43 of class C4, whereas SL1c includes the newly identified Ag43 ^C5^. Similarly, SL2a is present in Ag43 ^C2^ and SL2b in Ag43 ^C3^ passengers (Figure 4A). Overall, these findings showed that the Ag43 of C2-C5 classes were each associated with only a single SL2a, SL2b, SL1b, or SL1c subtype. The exceptions were the Ag43 ^C1^ and Ag43 ^C6^ which are both associated with the SL1a subtype.

As previously observed, two robust sequence subtypes were identified for each of the EJ and BL subdomains (Figure 4B,C). EJ1 was always found together with BL1, whereas EJ2 was with BL2. EJ1-BL1 were always present in Ag43 belonging to SF-I (Ag43 ^C1^ and Ag43 ^C3^), whereas EJ2-BL2 were in Ag43 belonging to SF-II (Ag43 ^C2^, Ag43 ^C4^, Ag43 ^C5^, and Ag43 ^C6^). Therefore, given that Ag43 ^C1^ and Ag43 ^C6^ both contained the SL1a subtype, their composition differed through their association with either the EJ1-BL1 or EJ2-BL2 subdomains. The EJ region reveals a remarkably conserved size of 85 amino acids regardless of the type 1 or 2 (supplementary Table S1). In contrast, BL1 was 143 amino-acids long compared to a length of 90 amino acids for BL2.

### 2.2. Identification of Conserved Domain Modules in Ag43

**Figure 5:**
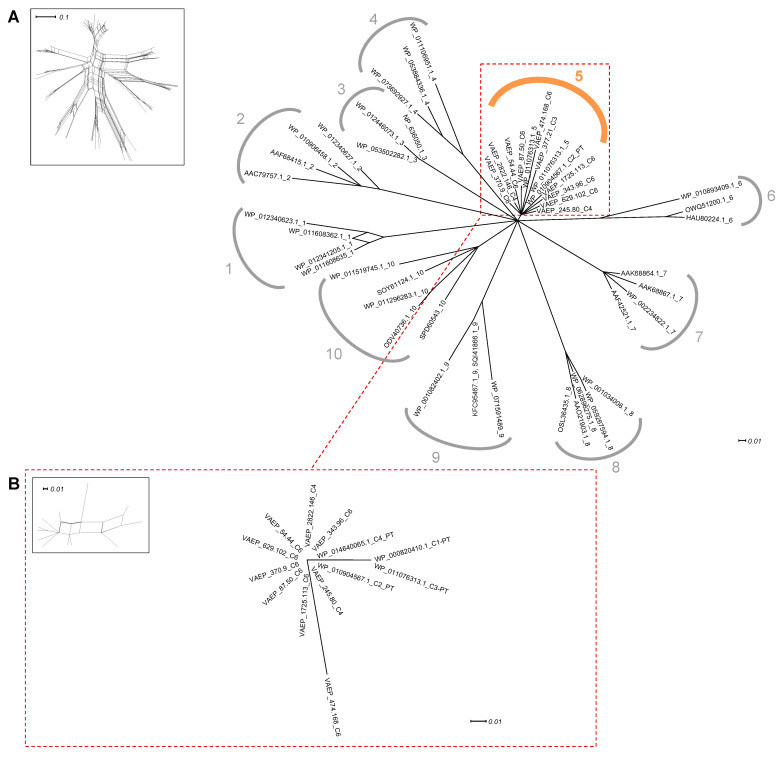
Phylogenetic analysis of the ESPR in Ag43. Trees were generated using SplitsTree; inserts represent the trees before applying bootstrapping and confidence threshold. (**A**) ESPR from Ag43 respective to the ten different AT ESPR clusters (numbered from 1 to 10) as previously reported, [24,33]. (**B**) Phylogenetic analyses of the EPSR from Ag43 only. For legibility, sequences that were identified but have not been included here are available in Table S1. Scale bars represent the evolutionary distance.

Given the discovery that the SL subdomains of the Ag43 passenger domains could be subdivided into six distinct evolutionary clusters, we sought to determine if the other Ag43 domains followed a similar evolutionary trajectory. Previously, it was demonstrated that the ESPR domain of all AT signal peptides could be divided into 10 clusters [24,33]. Our phylogenetic network analysis has further revealed that all Ag43 ESPRs belong to just a single cluster (cluster 5) (Figure 5; supplementary Table S1).

**Figure 6:**
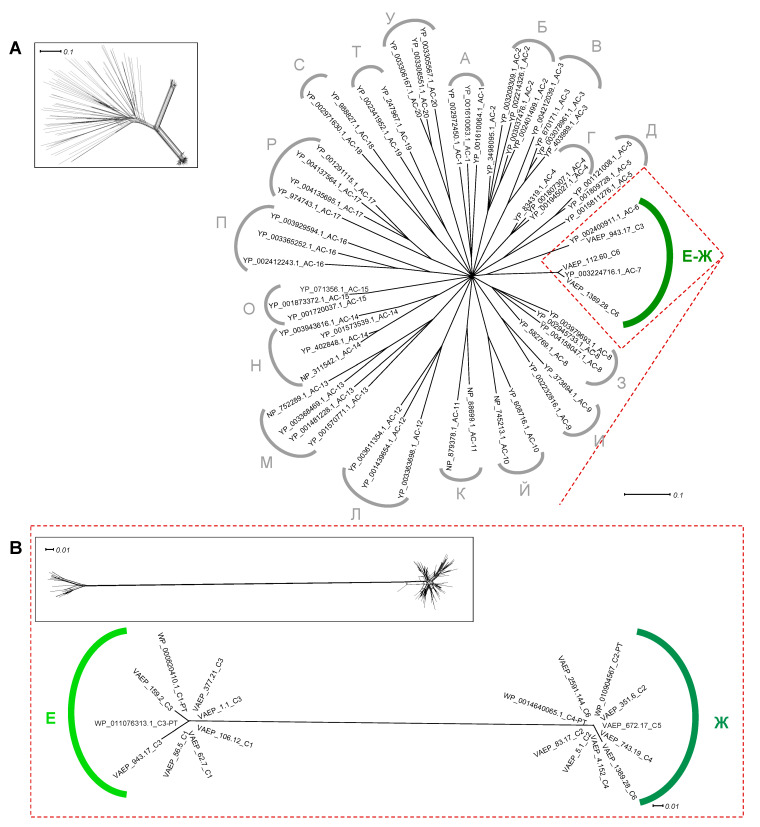
Phylogenetic analysis of the AC1 in Ag43. Trees were generated using SplitsTree; inserts represent the trees before applying bootstrapping and confidence threshold. (**A**) AC1 from Ag43 respective to the different clusters previously reported in ATs, namely from A to У (Cyrillic alphabet) [26]. (**B**) Phylogenetic analyses of the AC1 from Ag43 only. For legibility, sequences that were identified but have not been included here are available in Table S1. Scale bars represent the evolutionary distance.

Previous studies indicated that the AC1 subdomain of ATs could be divided into 20 main clusters (Figure 6A) [26], and that the AC1 domain of Ag43 and AIDA-I belong to E-Ж clusters. Our phylogenetic network analysis identified two robust sequence subtypes, i.e., (i) AC1^E^ that was only found among Ag43 belonging to SF-I (Ag43 ^C1^ and Ag43 ^C3^), and (ii) AC1^Ж^ that was present in Ag43 belonging to SF-II (Ag43 ^C2^, Ag43 ^C4^, Ag43 ^C5^ and Ag43 ^C6^) (Figure 6B). Consequently, AC1^E^ is associated with EJ1-BL1 subdomains, whereas AC1^Ж^ with EJ2-BL2 subdomains. While AC1^E^ displayed a conserved size of 114 amino acids, AC1^Ж^ was slightly longer with a size of 117 amino acids (supplementary Table S1).

**Figure 7:**
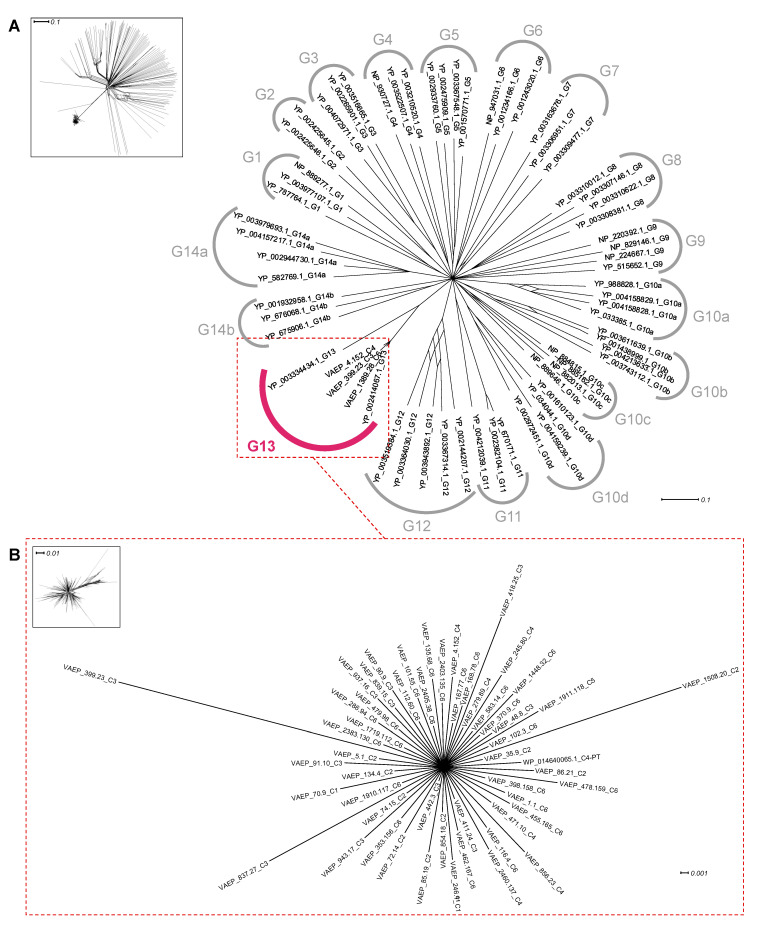
Phylogenetic analysis of the translocator in Ag43. Trees were generated using SplitsTree and the inserts represent the trees before applying bootstrapping and confidence threshold. (**A**) Translocator from Ag43 respective to the other translocators previously reported in ATs [34]. (**B**) Phylogenetic analyses of the Ag43 translocators. For legibility, sequences that were identified but have not been included here are available in Table S1. Scale bars represent the evolutionary distance.

The AT translocators were previously shown to have fourteen (G1-G14) distinct evolutionary clusters [34]. Phylogenetic analyses revealed that the 330 amino acid translocator domain of Ag43 was associated with the G13 cluster, which included the translocator domains of other SAATs such as AIDA-I (Figure 7; supplementary Table S1). Our analysis with the additional Ag43 sequences confirmed this result that the Ag43 translocators were monophyletic.

**Figure 8:**
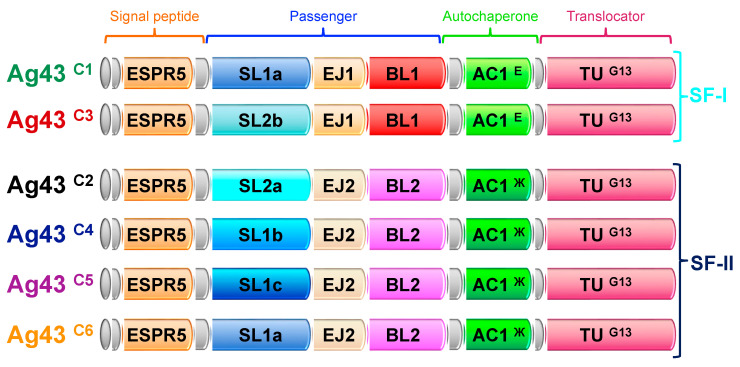
Diversity of Ag43 as revealed by in-depth phylogenetic analyses. Schematic representation of the variations in the modular architecture of Ag43 from C1 to C6 depicting the position of the ESPR, passenger encompassing the SL (stem L shape), EJ (elbow joining) and BL (bottom of L shape) subdomains, autochaperone (AC), and translocation unit (TU) regions, where the different types and subtypes of domains/subdomains are highlighted with colouring to show similarity as defined by the phylogenetic analyses (Figure 2, Figure 3, Figure 4, Figure 5, Figure 6 and Figure 7).

Our results demonstrate that the major sequence and functional variation across Ag43 homologues stems from passenger and AC1 subdomains, since the N-terminal (SP ESPR) and C-terminal region (translocator) belong to a single phylogenetic group (Figure 8). Remarkably, the EJ, BL, and AC1 subtypes appeared to be phylogenetically constrained, with Ag43 proteins containing EJ1-BL1-AC1 ^E^ domains belonging to SF-I and EJ2-BL2-AC1 ^Ж^ domains to SF-II. Nevertheless, it appears that four out of the six classes of Ag43 stem from four different SL subdomains (Figure 8); only SL1a is found in two classes of Ag43, namely C1 and C6. Both Ag43 ^C1^ and Ag43 ^C6^ are divided based upon SL1a having different associations with either EJ1-BL1-AC1 ^E^ or EJ2-BL2-AC1 ^Ж^, respectively. The finding that Ag43 SL domains account for much of the Ag43 diversity is consistent with the direct role in the Ag43 self-association mechanism that allows bacterial aggregation [21].

### 2.3. Distribution of Ag43 Paralogs within E. coli

Previously it was noted that *E. coli* can possess multiple copies of *agn43*. However, there was little understanding of whether these paralogous copies were gene duplications or the result of a single strain acquiring members of different Ag43 classes. To evaluate the distribution of *agn43* paralogs and investigate possible correlations between *agn43* alleles, we focused our analysis on complete genomes. All identified *agn43* sequences appeared to belong to bacteria from the phylum Proteobacteria, class Gammaproteobacteria, and order Enterobacterales, and almost exclusively to the family Enterobacteriaceae (supplementary Table S1). Further scrutiny of the data revealed that 99.6% of the *agn43* genes were present in the genus *Escherichia*, with exceptions including four *agn43* sequences found in *Klebsiella pneumoniae*, two in *Citrobacter* sp. And one in *Salmonella* sp. (supplementary Table S1). Within the *Escherichia* genus, 99.7% of the *agn43* sequences were found in *E. coli,* this includes the subspecies *Shigella,* where all members belong to the species *E. coli* [35]. Only a few *agn43* sequences were identified in *E. fergusonnii* (three sequences), *E. marmotae* or *E. albertii* (one sequence each) (supplementary Table S1). Overall, these data indicate that *agn43* is highly prevalent in *E. coli*.

**Figure 9:**
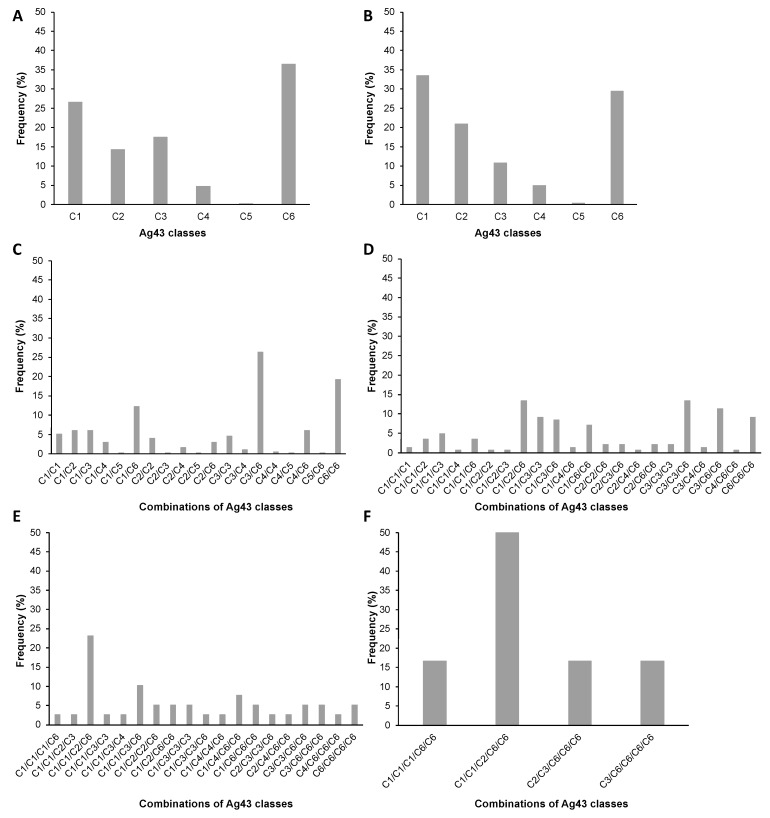
Prevalence and distribution for the different combinations of the 6 Ag43 classes in *Escherichia* genomes. Representation of the frequency of detection of the different Ag43 classes or combinations of classes in (**A**) all genomes encoding at least one copy of Ag43, (**B**) genomes encoding a single copy of Ag43, (**C**) genomes encoding two copies of Ag43, (**D**) genomes encoding three copies of Ag43, and (**E**) genomes encoding four copies of Ag43, and (**F**) genomes encoding four copies of Ag43 (supplementary Table S1).

Nevertheless, the occurrence of *agn43* was not ubiquitous in *E. coli*. It was present in only 67.2% of the 2173 *E. coli* genomes analysed (supplementary Table S1) and 65.8% of all *Escherichia* genomes. While the majority of the *Escherichia* genomes contained only one copy of *agn43* (62.4%), up to five *agn43* copies (0.4%) could be identified; 24.9% of the genomes harboured two copies; 9.6% harboured three copies; and 2.6% harboured four copies (supplementary Table S1). The genes encoding Ag43 were further categorised according to the six classes uncovered by our phylogenetic analyses. When considering all the Ag43 sequences identified in *Escherichia* genomes, C6, C1, and C3 appeared as the most prevalent Ag43 classes, reaching 36.5, 26.6, and 17.5%, respectively, whereas C2, C4, and C5 represented 14.3, 4.8, and 0.3% of the uncovered sequences (Figure 9A). However, when *Escherichia* genomes containing only a single *agn43* sequence were analysed separately, the relative prevalence was different: C1 (33.5%), C6 (29.5%), and C2 (20.9%), followed by C3, C4, and C5, ranging from 10.8 to 0.3%, respectively (Figure 9B).

Of the 21 possible combinations for two *agn43* alleles, 19 different combinations could be identified from genomic data. The coexistence of C3/C6 classes was the most frequent (26.3%), followed by C6/C6 (19.2%) and C1/C6 (12.2%), whereas the remaining combinations each represented less than 6% of the cases (Figure 9C). Interestingly, all genes encoding Ag43 ^C5^ representatives were only identified in the genomes harbouring one or a maximum of two copies of *agn43*. For genomes with three *agn43* copies, 22 out of the 56 theoretical combinations could be identified, where, in decreasing order, C1/C2/C6, C3/C3/C6, and C3/C3/C6 classes were the most prevalent (ranging from 13.4 to 11.3%); the remaining combinations each represented less than 10% of the cases (Figure 9D). When 4 *agn43* were present, only 19 combinations of classes could be observed from genomic data (out of the 126 mathematical possibilities), with the association of C1/C1/C2/C6 (23.1%) and C1/C1/C3/C6 (10.3%) appearing as the most frequent, as the following combinations dropped under 8% (Figure 9E). Finally, among the four combinations of 5 copies of *agn43* uncovered (out of the 256 theoretical possible combinations), C1/C1/C2/C6/C6 (50.0%) was the most frequent; the three others each had a 16.7% prevalence (Figure 9F). Taken together, it appears that when present in multicopy, genes encoding Ag43 ^C6^ were the most prevalent (found in 38.2% of cases), followed by Ag43 of classes C3 and C1 (22.5 and 22.4%, respectively); genes encoding Ag43 ^C4^ or Ag43 ^C5^ were the rarest (5.8% and 0.4%, respectively). Principal component analyses (PCA) revealed that genes encoding Ag43 ^C1^ were most significantly associated with genes of the same class (supplementary Table S1); a similar correlation was observed for genes encoding Ag43 ^C6^ or Ag43 ^C3^.

**Figure 10:**
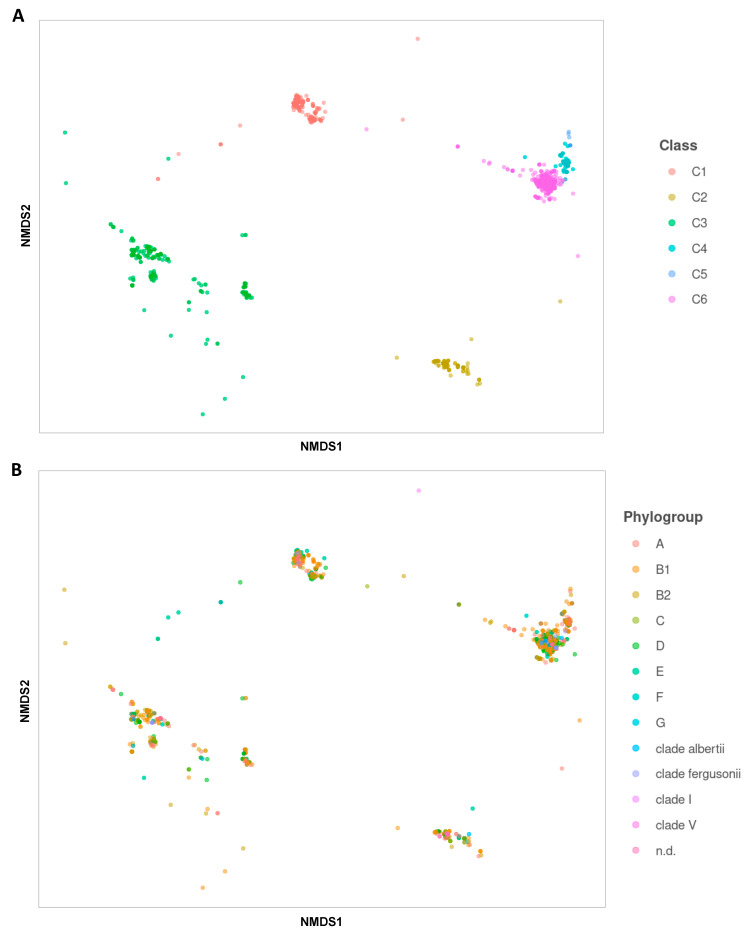
NMDS (non-metric multi-dimensional scaling) ordination plots of the relationships between *Escherichia* phylogroups and Ag43 classes. PERMANOVA (permutational multivariate analysis of variance) was performed to analyse the variance between genomic sequences and the phylogenetic tree of Ag43 passenger sequences. (**A**) NMDS highlighting the different Ag43 classes respective to dissimilarity matrix of Ag43 passenger sequences. (**B**) NMDS highlighting the different phylogroups respective to a dissimilarity matrix of Ag43 passenger sequences.

To assess the potential relationships between the dissimilarity matrix of Ag43 passenger sequences and Ag43 classes, as well as phylogroups, permutational multivariate analysis of variance (PERMANOVA) tests were performed [36,37]. In accordance with the phylogenetic analysis (Figure 3), a significant and strong separation between the different Ag43 classes was confirmed by non-metric multi-dimensional scaling (NMDS) ordination plot, especially between C1, C2, and C3 classes and C4/C5/C6, respectively (Figure 10A; *p*-value < 1 × 10^−4^ and R^2^ = 0.95). However, a statistically significant but non informative separation for the phylogroups could be evidenced by PERMANOVA (Figure 10B; *p*-value = 0.01 and R^2^ = 5 × 10^−4^).

**Figure 11:**
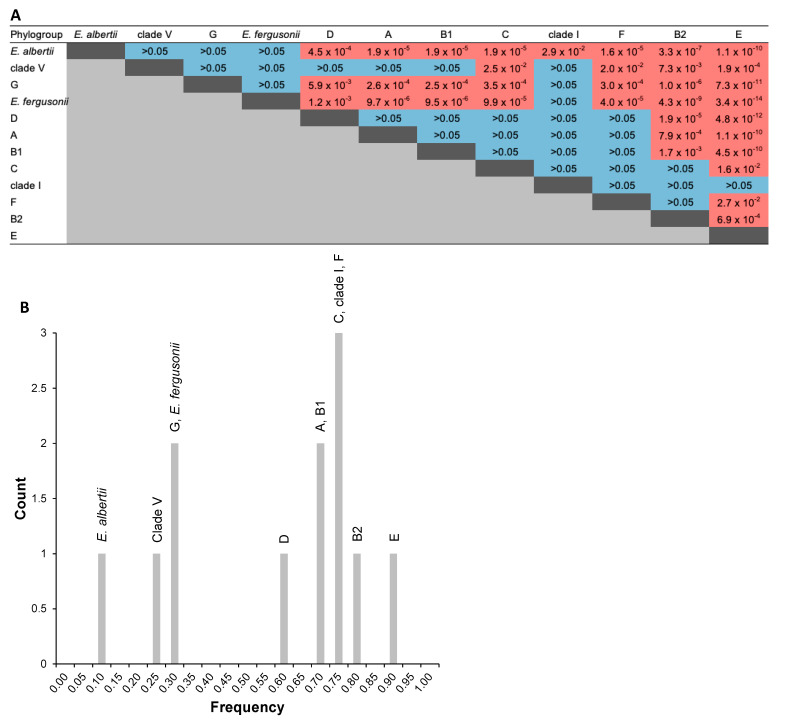
Statistical analyses of the relationships between the *Escherichia* phylogroups respective to presence or absence of *agn43*. (**A**) Table of the pairwise independence Fisher tests respective to the *Escherichia* phylogroups and the presence or absence of *agn43* in genomes. Pairwise independence (*p*-value > 0.05) or absence of independence (*p*-value < 0.05) are highlighted in blue and red, respectively. (**B**) Plot of the frequency count for the presence of *agn43* respective to the *Escherichia* phylogroups. Details of statistical analyses are available in supplementary Table S1.

Respective to the presence or absence of *agn43* in *Escherichia* genomes in relation to the phylogroups, statistical analyses following pairwise independence Fisher tests indicated an overall absence of independence (*p*-value = 1.5 × 10^−26^; supplementary Table S1). In summary, a dependence between the presence or absence of *agn43* was statistically evidenced for some but not all phylogroups. For example, compared to the absence of *agn43*, the presence of *agn43* was statistically more prevalent in E phylogroup compared to D (*p*-value = 4.8 × 10^−12^; Figure 11A) but not in B1 compared to D (*p*-value > 0.05; Figure 11A). Considering the frequencies (number of genomes containing *agn43* over total *Escherichia* genomes) (Figure 11B), four main groupings could be considered within the phylogroups, i.e., (i) E, where more than 90% of the *Escherichia* genomes contain *agn43*, (ii) D, A, B1, C, clade I, F, and B2, where more than 50% but less than 80% of the *Escherichia* genomes contain *agn43*, (iii) clade V, G, and *E. fergusonii* where more than 25% but less than 30% of the *Escherichia* genomes contain *agn43*, and (iv) *E. albertii* where less than 10% of the genomes contain *agn43*.

**Figure 12:**
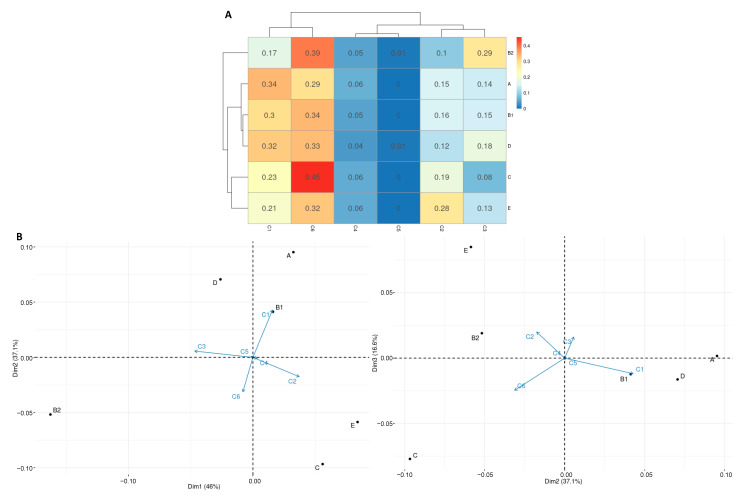
Statistical analyses of the relationships between the *Escherichia* phylogroups respective to the different Ag43 classes. (**A**) Heatmap indicating the ratio of the number of genomes per phylogroup and per Ag43 class. The dendrogram was calculated using an Euclidean distance matrix. Only phylogroups for which the absence of independence was statistically evidenced by pairwise independence Fisher tests (*p*-value < 0.05) were taken into account (*Escherichia* phylogroups F, G, cladeI, cladeV, *E. albertii*, and *E. fergusonii* were thus excluded, *p*-value > 0.05). (**B**) Plot of PCA based on the ratio of the number of genomes per phylogroup and Ag43 class over three dimensions. Detailed statistical analyses are available in supplementary Table S1.

Respective to the phylogroups and the different classes of Ag43, pairwise independence Fisher tests indicated an overall absence of independence (*p*-value = 1.3 × 10^−8^; supplementary Table S1). In detail, the absence of independence was only observed in a few cases, e.g., compared to Ag43 ^C3^, Ag43 ^C1^ was most prevalent in A phylogroup compared to B2. In the vast majority of cases (in 98% of all possible combinations), however, pairwise independence was observed following Fisher tests (supplementary Table S1). However, no pattern where a class of Ag43 would have been exclusively present in one or some phylogroups could be observed. Focusing on pairwise phylogroup and Ag43 class where an absence of independence was observed, a heatmap of the frequencies indicated that genes encoding Ag43 ^C4^ and Ag43 ^C5^ are poorly represented in each of the considered phylogroups (i.e., B2, A, B1, D, C, and E) and are homogeneously distributed (Figure 12A). By comparison, the Ag43 ^C1^ and Ag43 ^C6^ are well represented in all the considered phylogroups. Ag43 ^C3^ are essentially present in phylogroup B2, whereas Ag43 ^C2^ are essentially present in E phylogroup. Following a PCA, some strong correlations between some Ag43 classes and phylogroups could be evidenced (Figure 12B). A strong correlation was evidenced between the Ag43 ^C1^ and the phylogroups A, B1, and D; Ag43 ^C6^ is well correlated with C phylogroup, whereas Ag43 ^C2^ is well correlated with phylogroup E (Figure 12B).

## 3. Discussion

Previous phylogenetic analyses disclosed the existence of four distinct Ag43 phylogenetic classes that showed variation in their ability to promote aggregation and/or biofilm formation [20,29]. These different classes appear to have arisen by subdomain shuffling within the passenger, resulting in the following four combinations: (i) SL1-EJ1-BL1 corresponding to Ag43 ^C1^, (ii) SL2-EJ2-BL2 corresponding to Ag43 ^C2^, (iii) SL2-EJ1-BL1 corresponding to Ag43 ^C3^, and (iv) SL1-EJ2-BL2 corresponding to Ag43 ^C4^. Following extensive data mining of complete bacterial genomes to identify Ag43 homologues, we revealed two additional classes of Ag43, namely C5 and C6 (SL1-EJ2-BL2). We also disclosed a further subdivision of the SL subdomain into three subtypes (a, b, and c) for SL1, and two subtypes (a and b) for SL2 (Figure 4 and Figure 8). Interestingly, neither the association of SL1b, SL1c, or SL2a with EJ1-BL1-AC1 ^E^ nor the combination of SL2b with EJ2-BL2-AC1 ^Ж^ was observed. It can be either hypothesised that such combinations exist naturally but have not been sequenced, or they simply do not exist, which would suggest some genotypic, phenotypic, or structural constraints. While the ESPR and TU belong to the same monophyletic group, the AC1 appeared as the only one additional region displaying distinct phylogenetic clusters beyond the passenger. The strict association of AC1 ^E^ with EJ1-BL1 and AC1 ^Ж^ with EJ2-BL2 would suggest these AC1 variants are necessary for correct folding of these EJ-BL modules when they emerge from the translocator in the course of the secretion. Given the conservation of the translocator compared to the passenger, it was well accepted that this region underwent some domain shuffling [4,38]. Our studies extend this concept by showing for the first time that domain shuffling was even more complex, with regions of the ATs passenger undergoing different levels of transfer.

Phenotypically, Ag43 ^C1^ is the most characterised variant to date, with only a few investigations dedicated to Ag43 ^C2^ [39], Ag43 ^C3^, and, most recently, Ag43 ^C4^ [29]. The identification of the novel and prominent Ag43 ^C6^ variant, as well as Ag43 ^C5^, suggests further phenotypic characterisation is required to fully understand their ability to autoaggregate, adhere, and form a biofilm, as well as their propensity to co-interact with other Ag43 classes. Recent investigation of homotypic interactions of prototypical (PT) Ag43 variants has revealed that the autoaggregation kinetics were slower and the aggregates smaller for Ag43 ^C3-PT^ compared to other classes [29]. In light of our new findings, the difference between Ag43 ^C3-PT^ and other Ag43 classes is the SL2b subdomain, which is likely responsible for the weaker autoaggregation phenotype.

Heterotypic interactions were found to occur only for a limited number of combinations, which includes interactions between Ag43 ^C4-PT^ and Ag43 ^C1-PT^ as well as, to a lesser extent, between Ag43 ^C4-PT^ and Ag43 ^C3-PT^ [29]. This indicates Ag43 proteins with a SL1b-EJ2-BL2-AC1 ^Ж^ (Ag43 ^C4^) module may interact with Ag43 exhibiting a SL1a-EJ1-BL1-AC1 ^E^ pattern (Ag43 ^C1^) or, to a lesser degree, SL2b-EJ1-BL1-AC1 ^E^ pattern (Ag43 ^C3^). This suggests SL1b is more permissive to heterotypic interactions (two heterotypic interactions reported) than SL1a (one heterotypic interaction), SL2b (one heterotypic interaction), or SL2a (no heterotypic interaction). Common features of Ag43 ^C1^ and Ag43 ^C3^ are that they are the only Ag43 belonging to SF-I and exhibit a EJ1-BL1-AC1 ^E^ pattern. Possibly, the EJ1-BL1 module could play an indirect role in the heterotypic interaction by altering the angle between the SL and BL subdomains and the overall L-shaped structure, thus modulating the electrostatic and hydrogen bonds necessary for the interactions [21]. Considering Ag43 ^C1^ and Ag43 ^C6^ are the only proteins to harbour SL1a with different EJ-BL modules, experiments designed to probe homotypic interaction experiments with Ag43 ^C4^ could reveal the role of the SL subdomain over EJ-BL subdomains. Experimentally resolving the structures of Ag43 belonging to all six classes would greatly assist in deciphering the role of the different passenger subdomains in autoaggregation, and the potential for heterotypic associations between Ag43 variants [20,21].

While *agn43* is largely prevalent in *E. coli*, it did not appear to have an ubiquitous distribution as previously suggested [4]. Besides *E. coli*, *agn43* was previously identified in *Citrobacter rodentium* [18], but the present investigation is the first to report its presence in other *Escherichia* species, such as *E. albertii* or *E. fergusoni.* These *Escherichia* species can be present in various environments including the soil, water, and/or gastro-intestinal digestive tract where they may potentially interact [40]. Besides *Escherichia*, *agn43* was further identified in the *Klebsiella*, *Citrobacter*, and *Salmonella* genus. Bacterial species from all these genera are part of the normal intestinal microbiota of mammals, where they could interact. In *E. coli*, Ag43 was suggested to be more prevalent in pathogenic *E. coli* than in commensal strains, especially members of the SF-II [31]. However, observations of *agn43* in non-pathogenic strains do not support this hypothesis and may simply reflect the bias toward sequencing more pathogenic (compared to non-pathogenic) *E. coli* strains [18]. While a correlational analysis examining the potential relationship between pathogenic *E. coli* and the presence or absence of particular variants of *agn43* would have been of relevance, it is hindered by the lack of metadata regarding the pathotype, serotype/serogroup, and/or origin of the isolates for a large number of sequenced *E. coli* genomes we recovered. Additional work for comparison is hampered by the lack of robust and systematic data and information in the literature or databases despite relevant initiatives such as PATRIC (Pathosystems Resource Integration Center) [41]. Nonetheless, the relationships between the presence or absence of *agn43* as well as its different classes were statistically investigated respective to the *Escherichia* phylogroup [42]. In the genomes where *agn43* was present, a correlation between the Ag43 phylogenetic distances and phylogroup showed a significant but non informative correlation. This suggests *E. coli* phylogeny at least partially affects the distribution of Ag43 classes, with a significant difference in the frequency of Ag43 classes among different *Escherichia* clades. The most striking is probably the E phylogroup with the presence of *agn43* in 90% of *E. coli* genomes and the correlation with Ag43 ^C2^. As pathogens, *E. coli* from the E phylogroup are frequently DEC, especially ETEC, EHEC, including shigatoxin-encoding *E. coli* (STEC), enteropathogenic (EPEC), and enteroinvasive (EIEC) *E. coli* [43]. Ag43 ^C1^ is well correlated with A, B1, and D, which harbour *agn43* in about 60% of the *E. coli* genomes. As DEC, *E. coli* strains from these phylogroups can be EIEC, EPEC, ETEC, EHEC/STEC, or enteroaggregative *E. coli* (EAEC) [43]. Though, it must be stressed that these different phylogroups are not strictly associated with enteropathotypes, since numerous strains can be commensal, pathobiont, or opportunistic pathogenic *E. coli*, such as extra-intestinal pathogenic *E. coli* (ExPEC) [44].

The role of bacterial autoaggregation has been questioned on numerous occasions in the scientific literature, including regarding contribution to infections, antibiotic resistance, or biofouling in the environment [9,45]. Previously, it was demonstrated that Ag43 promotes surface colonisation and sessile development following sedimentation of cell aggregates [18,20]. In UPEC, Ag43 participates in urinary tract colonisation and contributes to chronic infections [46,47]. However, there is no evidence to support a significant contribution to intestinal colonisation [48]. An additional function in bacterial physiopathology was proposed where Ag43 would promote uptake and survival in polymorphonuclear neutrophils [49], but its significance and relevance remain to be thoroughly evaluated [18]. Clearly, the full picture of the contribution of Ag43 to *E. coli* biology still requires further in-depth investigations. Nevertheless, the effects of the different classes of Ag43 on these phenotypes remain to be evaluated. Our new understanding of the diversity of Ag43 provides a novel and rational framework for investigating its role in *E. coli* physiology, ecophysiology, and physiopathology, particularly its involvement in the organisation of *E. coli* communities in complex ecosystems such as the intestinal microbiota of human or animals.

## 4. Materials and Methods

### 4.1. Data Mining for Identification of Ag43 Protein Sequences

Genomic data were retrieved from the NCBI assembly database [50]. Ag43 homologues were recovered using a personal database of 106 protein sequences corresponding to the passenger domains and their assignment to the four classes of Ag43 recently uncovered [29]. BLASTX analysis (E-value threshold 1 × 10^−100^ and low-complexity filter disabled) was carried out with BLAST+ command-line tools version 2.7.1 [51]. All HSP (high scoring pairs) alignments with more than 90% similarity and encompassing more than 400 amino acids were considered for the following studies. In the first instance, genome sequences have been downloaded using *Escherichia coli* as keywords. According to the degree to which they have been assembled, such sequences corresponded to genomes, chromosomes, contigs, or scaffolds. These DNA sequences were used to extract the complete Ag43 protein sequences without considering annotation information from genomic data. Such an approach allowed us to highlight mispredicted translation initiation sites (TISs), errors introducing frameshift or premature stop codon, and insertion of DNA sequence, including some mobile elements disrupting the CDS (coding DNA sequence). All incomplete sequences due to the events mentioned above were collectively considered as pseudogenes. In order to recover Ag43 homologues from *Enterobacteriaceae* other than *E. coli*, sequences from the genus *Escherichia* but not referenced as *E. coli* species and *Enterobacteriaceae* sequences, excluding genus *Escherichia*, have been also extracted from the NCBI assembly database, respectively. Finally, BLASTX analysis was performed against the NCBI assembly database excluding *Enterobacteriaceae*, in order to recover Ag43 homologues in the Kingdom Bacteria other than the ones identified here above. The detailed bioinformatic analysis is provided as supplementary material (File S1) and Perl scripts developed for this study are available on GitHub (https://github.com/ivwawrzy/Ag_43, accessed on 5 March 2023).

Excluding pseudogenes, Ag43 CDS recovered by this approach were clustered with a threshold of 98% using Cd-hit software [52,53], and a reference sequence from each cluster was kept to generate a representative Ag43 dataset used for the phylogenetic studies as described below. Considering the modular architecture of Ag43, the Ag43 protein sequences were split as follows. For the identification of the SP, the sequences were submitted to SignalP v5.0 [54]. Using the InterPro (IPR) database [55], the ESPR (IPR024973) was identified using InterProScan v5.22 [56]. The passenger was identified following structural alignment as explained below and the SL, EJ, and BL subdomains were identified from three-dimensional (3D) structures using UCSF Chimera visualisation [19] as previously described [21,29]. The AC1 domains were identified based on a structural alignment using Phyre v2.0 for automated modelling [22] and UCSF Chimera for 3D structure visualisation as previously described [26]. The translocators were identified using the twin HMM (Hidden Markov Model) strategy for AT detection [34]. From full length Ag43, the sequences were then split into modules corresponding to the ESPR, passenger, SL, EJ, BL, AC1, and translocator, also called translocation unit (TU).

### 4.2. Phylogenetic Network Analysis of Protein Sequences

For multiple sequence alignment of the full length A43 including the (i) passenger and its subdomains, (ii) AC1, and (iii) translocator, a special mode of T-Coffee [57,58,59] was used to incorporate structural information, i.e., Expresso [60], which is an extension of 3D-Coffee where structure based alignment is used as a template [61]. Thus, the amino acid sequences were aligned using solved structures from the protein data bank (PDB) as templates [62,63], namely (i) the passenger from Ag43 (PDB: 4KH3), (ii) AC1 from IcsA (PDB: 3ML3), and (iii) the translocation units comprising both the α-linker and β-barrel from AIDA-I (PDB: 4MEE) [23], EspP (PDB: 3SLJ) [64], EstA (PDB: 3KVN) [65], Hbp (PDB: 3AEH) [66], and NalP (PDB: 1UYN) [67], respectively. The relatedness among protein sequences was studied using a Neighbor-Net phylogenetic network approach with SplitsTree v4.14.8 using the Hamming uncorrected-P distance [68]. The most relevant clusters, i.e., monophyletic groups or clades, were identified and selected based on splits or branches showing bootstrap values above 90% over 1000 pseudo-replicates.

### 4.3. Statistical Analyses

*Escherichia* phylogroups were identified using in the ClermontTyping software [42]. To test independence between phylogroups and the presence or absence of *agn43*, pairwise independence Fisher tests were performed using contingency tables with rcompanion R (v 4.1.2, R Core Team) package [69,70]. The same tests were performed to evaluate the independence between phylogroups and the different Ag43 classes, whenever *agn43* was present in single or multicopy in genomes. PCA (principal component analyses) were performed with the ade4 R package, where the values were centred by mean, not scaled [71].

To analyse the variance between genomic sequences, a PERMANOVA (permutational multivariate analysis of variance) was performed on the maximum likelihood distance matrix obtained from the phylogenetic tree of Ag43 passenger sequences. The distance matrix was computed with a maximum likelihood approach and LG model with the phangorn v2.9.0 R package [37]. Two sources of variations were used to fit the linear model: phylogroups and classes with interaction. The distance matrix and the PERMANOVA was performed, respectively, with the phangorn [37] and the adonis2 command of vegan [36] R packages. A Nonmetric Multidimensional Scaling (NMDS) ordination method fitted on Phylogroup and Class variables was used to visualise variability between them.

## Data Availability

Data are provided as supplementary materials. Additional data that support the findings of this study can be made available upon reasonable request from the corresponding authors.

## Supplementary Materials

Supporting information can be downloaded at https://www.mdpi.com/article/10.3390/ijms24065500/s1.
