# Supplementary material for: Genome-Wide Analysis of Antigen 43 (Ag43) Variants: New Insights in Their Diversity, Distribution and Prevalence in Bacteria
Source: Int J Mol Sci. 2023 Mar 13;24(6):5500. doi: 10.3390/ijms24065500 (PMC10058404; doi:10.3390/ijms24065500)
Supplement: Supplementary file 1 [file ijms-24-05500-s001.zip › FileS1.pdf]

**File S1: Description of Perl scripts developed to ensure identification and extraction of Ag43 sequences from NCBI genomic data.**

The BLATX analysis was carried out using Blastx_analysis.pl script (all scripts mentioned here below are available on GitHub at LMGE-IHP/Ag43 repository). It uses as argument a directory containing FASTA sequence files extracted from the genbank database. The BLATX analysis is done with the following command line: blastx -db ~/blastdb/domaine_passager -evalue 1e-100 -out $fichier_sortie -outfmt 7”. The outup file name was automatically generated with the FASTA file name substituting the .fna extension with _llastx.

BLATX result were parsed using parsing_blastx_result.pl. This script used two arguments: The directory containing BLATX result files generated by Blastx_analysis.pl script and an output file. In this file, BLATX results with an alignment length superior of 410 nt and more than 90 percent of similarity for C1 and C2 and 480 nt and more than 90 percent of similarity for C3 and C4 respectively were conserved in csv format. Begin and end positions of database sequences producing significant result were also extracted.

Such positions have been then used to do extraction of passenger amino acids sequence from respective FASTA sequence files (.fna extension). To ensure this specific sequence extraction, passenger_extraction.pl script was developed. It uses as first argument output file produced by the parsing_blastx_result.pl script and as second one as output file to recover passenger sequence in a FASTA format. This script must be run in the directory containing the FASTA sequences of the studied genomes.

In addition, complete_protein_Ag43_sequence_extraction_csv.pl script producing a csv file was developed to extract complete Ag 43 protein sequence using FASTA sequence files (.fna extension). Such file use output file produced by the parsing_blastx_result.pl. Furthermore, some annotation data from genbank files were also extracted using downloaded .gbff files. Thus, potential annotation mistakes or annotation omissions were also retrieved. All extracted data were stored in a csv file. This script is run in the directory containing the file generated by parsing_blastx_result.pl and all .fna and .gbff files respectively. The name of output file is given as second argument of the command line.

All passenger domains sequences were firstly sorted by Ag43 type using the parsing_type_Ag43.pl script 5. This script used an option (-t) to specify the class of Ag43

sequences to extract (C1, C2, C3 or C4) and two arguments. The first one is the FASTA file produced by passenger_extraction.pl script and the second one is the name of output file. Each Ag43 class were then clustered independently with a threshold of 98 % using Cd-hit software (Li et al. 2006, Li et al. 2006). To ensure easier exploitation of data produced by Cd-hit software, a series of scripts has been developed. The sequence_numbering_for_cdhit.pl script numbers each sequence submitted to the Cd-hit processing. The first argument of this script is file produced by the parsing_type_Ag43.pl script and the second one the name of the output file. The add_passenger_cluster_number.pl script allows for each sequence in FASTA file generated by produced by the parsing_type_Ag43.pl to add the cluster number on the comment line. This script used three arguments: The “.clstr” file generated by the Cd-hit analysis, the file containing all processed sequences and the name of the ouput file. The t Option allows to specify the Ag43 class (C1, C2, C3 or C4) processed. Finally, files generated by this analysis are concatened and file result is then used as the first argument of the add_complete_cluster_number.pl script to add cluster information to the csv file containing the complete Ag43 complete sequences (file produced by the complete_protein_Ag43_sequence_extraction_csv.pl script use as second argument).
